# von Willebrand Factor-Rich Platelet Thrombi in the Liver Cause Sinusoidal Obstruction Syndrome following Oxaliplatin-Based Chemotherapy

**DOI:** 10.1371/journal.pone.0143136

**Published:** 2015-11-18

**Authors:** Naoto Nishigori, Masanori Matsumoto, Fumikazu Koyama, Masaki Hayakawa, Kinta Hatakeyayama, Saiho Ko, Yoshihiro Fujimura, Yoshiyuki Nakajima

**Affiliations:** 1 Department of Surgery, Nara Medical University, Kashihara, Japan; 2 Department of Blood Transfusion Medicine, Nara Medical University, Kashihara, Japan; 3 Department of Diagnostic Pathology, Nara Medical University, Kashihara, Japan; National Cerebral and Cardiovascular Center, JAPAN

## Abstract

Oxaliplatin-based chemotherapy is widely used to treat advanced colorectal cancer (CRC). Sinusoidal obstruction syndrome (SOS) due to oxaliplatin is a serious type of chemotherapy-associated liver injury (CALI) in CRC patients. SOS is thought to be caused by the sinusoidal endothelial cell damage, which results in the release of unusually-large von Willebrand factor multimers (UL-VWFMs) from endothelial cells. To investigate the pathophysiology of CALI after oxaliplatin-based chemotherapy, we analyzed plasma concentration of von Willebrand factor (VWF) and the distribution of VWFMs in CRC patients. Twenty-three patients with advanced CRC who received oxaliplatin-based chemotherapy with (n = 6) and without (n = 17) bevacizumab were analyzed. CALI (n = 6) and splenomegaly (n = 9) were found only in patients who did not treated with bevacizumab. Plasma VWF antigen (VWF:Ag) and serum aspartate aminotransferase (AST) levels increased after chemotherapy only in patients without bevacizumab. VWFM analysis in patients who did not receive bevacizumab showed the presence of UL-VWFMs and absence of high molecular weight VWFMs during chemotherapy, especially in those with CALI. In addition, plasma VWF:Ag and AST levels increased after chemotherapy in patients with splenomegaly (n = 9), but not in patients without splenomegaly (n = 14). Histological findings in the liver tissue of patients who did not receive bevacizumab included sinusoidal dilatation and microthrombi in the sinusoids. Many microthrombi were positive for both anti-IIb/IIIa and anti-VWF antibodies. Plasma UL-VWFM levels might be increased by damage to endothelial cells as a result of oxaliplatin-based chemotherapy. Bevacizumab could prevent CALI and splenomegaly through inhibition of VWF-rich platelet thrombus formation.

## Introduction

Colorectal cancer (CRC) is the second most common cancer and fourth most common cause of cancer-related death worldwide [1]. Systemic chemotherapy for CRC with modern chemotherapeutic and biological agents has undergone dramatic advances over the last decade; the tumor response rate is now as high as 80%. The most commonly used systemic chemotherapy for advanced CRC is a combination of 5-fluorouracil (5-FU), folinic acid, and either oxaliplatin (FOLFOX) or irinotecan (FOLFIRI) [2, 3]. Recently, monoclonal antibodies against vascular endothelial growth factor (VEGF-A) such as bevacizumab and epidermal growth factor receptor (EGFR-A) such as cetuximab and panitumumab have also contributed to improvements in tumor response rate and survival [4].

Although oxaliplatin-based chemotherapy has benefited patients with CRC, chemotherapy-associated liver injury (CALI), including sinusoidal obstruction syndrome (SOS), has been observed after cytotoxic therapy. Histopathological findings associated with oxaliplatin-induced sinusoidal injury are very similar to those seen in SOS [5]. Rubbia-Brandt et al [6]. reported that sinusoidal dilatation in post-chemotherapy liver resection specimens was strongly associated with oxaliplatin use. In previous studies, SOS following oxaliplatin-based chemotherapy caused splenomegaly and thrombocytopenia [7, 8]. One randomized clinical study showed that bevacizumab in addition to conventional chemotherapy prolonged survival in patients with metastatic CRC compared to conventional chemotherapy alone [9]. The combination of bevacizumab and FOLFOX was associated with a lower incidence of SOS and thrombocytopenia than for FOLFOX alone [10]. In addition, bevacizumab was reported to have a protective effect in the liver of patients treated with oxaliplatin-based chemotherapy for CRC [11].

We have previously reported that plasma ADAMTS13 activity (ADAMTS13:AC) was reduced in patients with SOS, formerly called hepatic veno-occlusive disease (VOD), after hematopoietic stem cell transplantation (SCT) [12]. Subsequently, we found that high molecular weight von Willebrand factor (VWF) multimers (H-VWFMs) were defected during the early post-SCT stage and the number of unusually large VWF multimers (UL-VWFMs) was elevated prior to SOS onset [13]. VWF is synthesized in vascular endothelial cells and released into the plasma as UL-VWFM, the most active form with respect to platelet interaction [14]. In the normal circulation, UL-VWFMs are rapidly degraded into smaller VWFMs by ADAMTS13 under high shear stress conditions [15]. ADAMTS13:AC deficiency increases plasma levels of UL-VWFMs, leading to platelet thrombi under high shear stress conditions and resulting in thrombotic thrombocytopenic purpura (TTP). ADAMTS13:AC is lower in individuals with congenital ADAMTS13 gene mutations (Upshaw-Schulman syndrome) and individuals with acquired autoantibodies against ADAMTS13.

In this study, we investigated plasma VWF in patients with advanced CRC receiving oxaliplatin-based chemotherapy to determine the pathophysiology of CALI. Furthermore, we confirmed the relationship between platelet thrombi and liver injury based on pathological and immunohistochemical findings in liver tissue. We also investigated the protective effect of bevacizumab against liver injury via VWF.

## Patients, Materials and Methods

### Patients

Twenty-nine patients with CRC who received oxaliplatin-based chemotherapy between February 2011 and August 2013 at Nara Medical University Hospital were included in this study. These patients were treated with the following 3 oxaliplatin-based regimens with or without bevacizumab/panitumumab: 1) 5-FU and folinic acid plus oxaliplatin (FOLFOX6), 2) capecitabine plus oxaliplatin (CapeOX), or 3) S-1 plus oxaliplatin (SOX). These treatments comprised adjuvant chemotherapy after radical surgery in 14 patients, chemotherapy for unresectable metastatic CRC in 11 patients, and neoadjuvant chemotherapy to treat advanced CRC in 5 patients. Six patients were excluded from this study for the following reasons: tumor progression (n = 3), self-discontinuation (n = 2), and adverse effects of chemotherapy (n = 1).

Ultimately, 23 CRC patients were analyzed in this study (Table 1): 12 patients who received adjuvant chemotherapy, 6 patients who received chemotherapy for unresectable metastatic CRC, and 5 patients who received neoadjuvant chemotherapy. Of these, 13 were treated with an oxaliplatin-based regimen only, 4 were treated with an oxaliplatin-based regimen plus panitumumab, and 6 were treated with an oxaliplatin-based regimen plus bevacizumab (Table 1). CALI was diagnosed based on the following 2 criteria: 1) serum alanine aminotransferase (ALT), aspartate aminotransferase (AST), or total bilirubin (T-Bil) more than 2 times the upper limit of normal range at our hospital (ALT >70 IU/mL, AST >70 IU/mL, T-Bil 2.0 mg/dL) and 2) no other explanation for CALI.

**Table 1:** Clinical characteristics, chemotherapy regimen, response of chemotherapy and spleen size ratio.

patient	age	sex	primary lesion	TNM stage	purpose	chemotherapy regimen	response #	spleen size ratio ##	CALI	SOS grade
1	71	M	rectum	IVA	neoadjuvant	FOLFOX6+Pan	NC	0.83		
2	54	F	rectum	IIC	adjuvant	SOX	no rec.**	0.92	yes	
3	46	F	rectum	IIIC	adjuvant	SOX	no rec.**	0.95		
4	58	M	sigmoid colon	IVA	neoadjuvant	FOLFOX6+Pan	PR	0.96	yes	Grade 2
5	56	F	rectum	IVA	UMCRC*	FOLFOX6	NC	1.07		
6	41	F	rectum	IIC	adjuvant	CapeOX	no rec.**	1.12	yes	
7	53	M	rectum	IVB	neoadjuvant	FOLFOX6+Pan	PR	1.19		Grade 2
8	56	M	rectum	IVA	adjuvant	CapeOX	no rec.**	1.2		
9	57	M	rectum	IVA	neoadjuvant	FOLFOX6+Pan	PR	1.52		
10	62	M	rectum	local rec.**	adjuvant	SOX	no rec.**	1.58		
11	48	M	rectum	IIIB	adjuvant	CapeOX	no rec.**	1.59	yes	Grade 0
12	73	F	transverse colon	peritoneal rec.	UMCRC*	CapeOX	NC	1.61		
13	50	F	descending colon	IVB	adjuvant	CapeOX	no rec.**	1.67		
14	62	F	sigmoid colon	IIC	adjuvant	CapeOX	no rec.**	1.68		
15	68	M	sigmoid colon	IVA	adjuvant	CapeOX	no rec.**	1.96		
16	69	M	rectum	IVA	adjuvant	CapeOX	no rec.**	2.3	yes	
17	59	M	rectum	IIIC	adjuvant	CapeOX	no rec.**	2.93	yes	
18	38	M	sigmoid colon	IVA	adjuvant	FOLFOX6+Bev	no rec.**	1.00		
19	57	M	sigmoid colon	IVB	UMCRC*	FOLFOX6+Bev	PR	1.02		
20	63	M	descending colon	IVB	UMCRC*	FOLFOX6+Bev	PR	1.07		
21	61	F	rectum	IVA	neoadjuvant	CapeOX+Bev	NC	1.09		Grade 1
22	67	F	transverse colon	IVB	UMCRC*	FOLOFOX6→FOLFOX6+Bev	NC	1.17		
23	71	M	sigmoid colon	IVB	UMCRC*	FOLFOX6+Bev	PR	1.37		

^#^ tumor response was evaluated by Response Evaluation Criteria in Solid Tumors (RECIST). (response)

^##^ the spleen size ratio was the ratio of spleen size after chemotherapy to those before chemotherapy (spleen size ratio)

* unresectable metastatic colorectal cancer (UMCRC)

** recurrence (rec.)

Patients 18 through 23 were treated without bevacizumab.

This study was performed with the permission of the ethics committee of Nara Medical University and complied with the principles expressed in the Declaration of Helsinki. Written informed consent was obtained from each patient.

### Blood sampling

Plasma samples were collected from CRC patients at 5 or 6 time points during chemotherapy every month, starting at baseline before chemotherapy (month 0) for 4 or 5 months after the initiation of chemotherapy. Chemotherapy was suspended when the patients underwent liver section for metastatic liver cancer. These samples were taken before the starting chemotherapy. Blood was collected in plastic tubes containing 1/10 volume of 3.8% sodium citrate. The plasma was separated by centrifugation at 3,000 g for 15 minutes at 4°C. Aliquots were stored at -80°C until use.

### Assays of VWF antigen, activity, and ADAMTS13 activity

Plasma VWF:Ag levels were measured by sandwich ELISA using a rabbit anti-human VWF polyclonal antiserum (DAKO, Denmark) [16]. The value obtained from normal individuals (n = 20) in this assay was 102 ± 33% [16]. To determine the VWF activity, the collagen binding activity of plasma VWF (VWF:CB) was measured using a commercially available kit (VWF-CBA ELISA, PROGEN Biotechnik GmbH, Heiderberg, Germany) according to the manufactures’s instructions. ADAMTS13:AC was determined using a commercially available chromogenic act-ELISA kit (Kainos Laboratories Inc., Japan) [17]. The value obtained for normal individuals (n = 55) in the act-ELISA was 99 ± 22%.17 The value of 100% was defined as the amount of VWF:Ag and ADAMTS13:AC in pooled normal human plasma (NP). To evaluate the both levels of VWF and ADAMTS13, the ratio of VWF:CB to ADAMTS13:AC was calculated in this study.

### VWF multimer analysis

Multimer analysis of plasma VWF was essentially performed according to the method of Ruggeri and Zimmerman [18], with modifications as reported by Warren et al [19]. The lower gel consisted of 1% agarose (SeaKem^®^ Gold Agarose, Lonza, Rockland, ME, USA) and 10% glycerol dissolved in 50 mmol/L phosphate buffer (pH 8.8) with 0.1% sodium dodecyl sulfate (SDS). The upper gel was prepared with 0.8% agarose (SeaKem^®^ HGT Agarose, Cambrex, Rockland) dissolved in 370 mmol/L phosphate buffer (pH 6.8) with 0.1% SDS. The electrophoresis buffer consisted of 50 mmol/L Tris-glycine buffer (pH 8.3) containing 0.1% SDS. The experimental conditions including western blotting with luminographic detection were as previously described by Budde et al [20]. Multimers were classified as low molecular weight (corresponding to bands 1–5 in the VWFM analysis), intermediate molecular weight (bands 6–10), and H-VWFM (bands >10) [21]. High molecular weight bands that were not detected in NP were defined as UL-VWFMs.

### Measurement of spleen size

Computed tomography (CT) to measure spleen size was performed on 2 occasions, before chemotherapy and 3–5 months after the start of chemotherapy. Spleen size was determined using the outline of the spleen on each axial CT image (5 mm section thickness). The sum of the area of the spleen in each section, taking into account slice thickness, was calculated by tracing the contour of the spleen using an electronic free curve tool provided by the software (Synapse^®^, Tokyo, Japan). Splenomegaly was defined as a 50% increase over baseline in spleen size at 3–5 months after the start of chemotherapy [7]. The spleen size ratio was the ratio of spleen size after chemotherapy to those before chemotherapy. Therefore, spleen size ratio ≥ 1.5 was defined as having splenomegaly.

### Immunohistochemistry in liver specimens

Serial sections were stained by hematoxylin and eosin (H. E.) to identify the basic constituents of thrombi. Immunohistochemistry staining was performed to clarify the distribution of platelets and fibrin. Formalin-fixed, paraffin-embedded tissues were cut into 5-μm sections, deparaffinized, and rehydrated in a graded series of ethanol. Antigen retrieval was done by heating tissue sections using Target Retrieval Solution at pH 6.0 (DAKO Japan, Kyoto, Japan). To block endogenous peroxidase activity, sections were immersed in a 0.3% solution of hydrogen peroxide in absolute methanol for 5 minutes at room temperature and washed 3 times with fresh PBS for 5 minutes each. Anti-IIb/IIIa (Affinity Biologicals, South Bend, Canada), anti-VWF (DAKO), anti-fibrin (Accurate Chemical and Scientific Corporation, Westbury, NY, USA) antibodies were added to the sections, which were incubated overnight at 4°C. Sections were washed in PBS for 5 minutes thrice. We then used the ImmPRESS reagent kit, Mouse/HRP, or Rabbit/HRP (VECTOR) for VWF and fibrin, and anti-Sheep IgG (Jackson ImmunoResearch, West Grove, PA, USA) for IIb/IIIa according to the instructions of the manufacturer. Reaction products were visualized with 3,3´-diaminobenzidine (DAB) tetrahydrochloride. The sections were counterstained with hematoxylin, dehydrated in ethanol, cleared in xylene, and coverslipped.

Consecutive slices of non-tumor liver parenchyma were reviewed. The severity of sinusoidal congestion was graded from 0 to 3 as proposed by Rubbia-Brandt et al.:6 grade 0 = absent, grade 1 = mild (one-third of the lobule is affected), grade 2 = moderate (two-thirds of the lobule are affected), and grade 3 = severe (the entire lobule is affected).

### Statistical analysis

Statistical analysis was performed using the Mann-Whitney U test to compare the differences between groups, and Wilcoxon's signed rank test to compare the differences among any time points in each group. P<0.05 was considered statistically significant. Data are expressed as median (minimum-maximum). Statistical analysis was performed using GraphPad Prism software, version 6.01 (GraphPad Software, San Diego, CA, USA).

## Results

### Comparison between patients who received or did not receive bevacizumab

**Fig 1:**
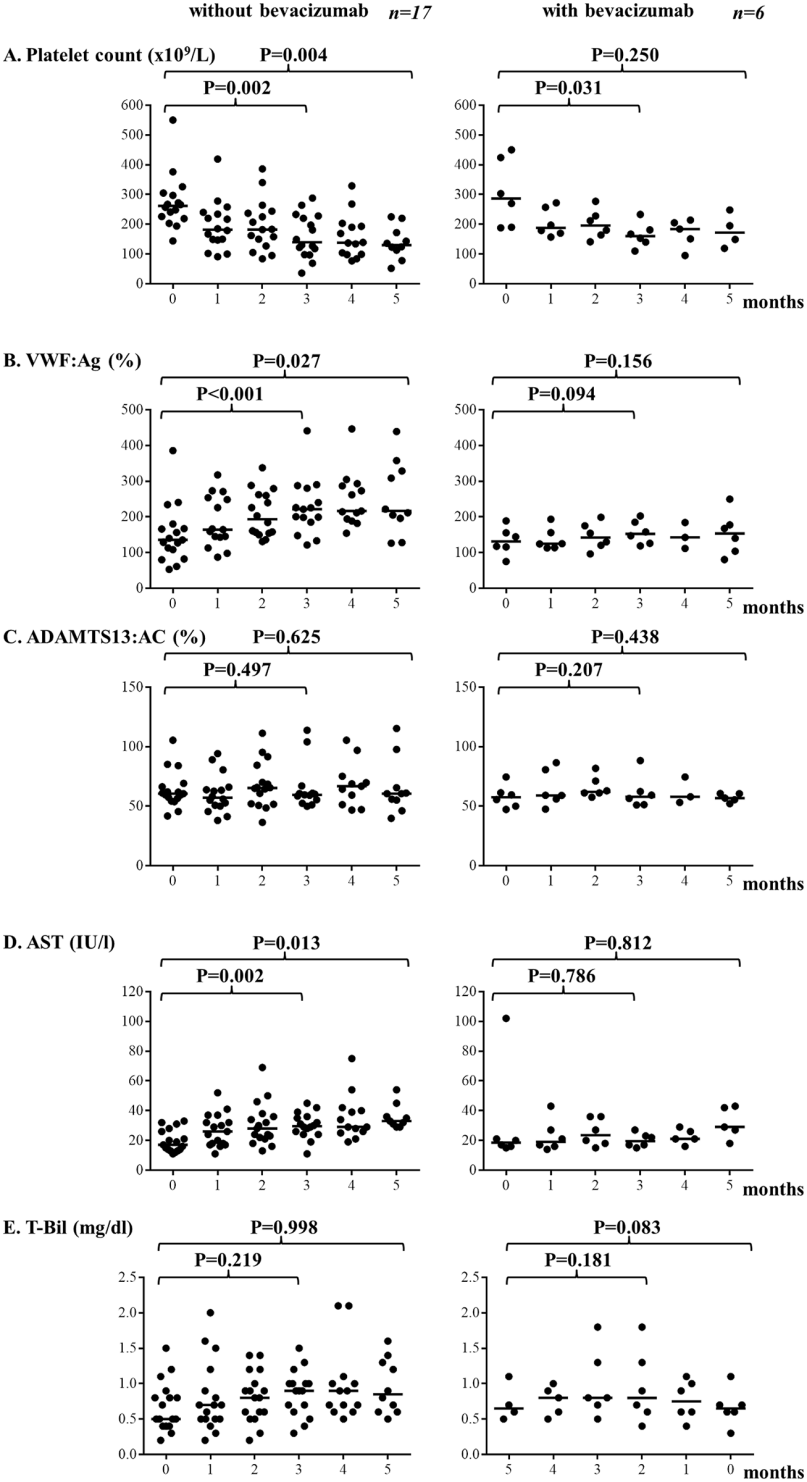
Comparison of platelet count, VWF:Ag, ADAMTS13:AC, AST, and T-Bil between patients treated with and not treated with bevacizumab. (A) Platelet counts decreased until months 3 as the number of chemotherapy cycles increased in both patients who received and did not receive bevacizumab. However, platelet counts in patients not treated with bevacizumab decreased much less in patients who received bevacizumab in months 5. (B) Plasma levels of VWF:Ag increased as the number of chemotherapy cycles increased in patients not treated with bevacizumab, but did not change in patients treated with bevacizumab. (C) Plasma levels of ADAMTS13:AC were unchanged in both groups. (D) Serum AST levels increased as the number of chemotherapy cycles in patients who did not receive bevacizumab, but they were unchanged in patients with bevacizumab. (E) Plasma levels of T-Bil did not change significantly in either group. VWF:Ag von Willebrand factor antigen, ADAMTS13:AC ADAMTS13 activity, AST Aspartate transaminase, T-Bil total bilirubin.

We classified patients into 2 groups: oxaliplatin-based chemotherapy with (n = 6) bevacizumab and without (n = 17) bevacizumab. As shown in Fig 1A, platelet counts decreased as the number of chemotherapy cycles increased among patients receiving bevacizumab (P = 0.002 at 3 months, P = 0.004 at 5 months) and not receiving bevacizumab (P = 0.031 at 3 months, P = 0.250 at 5 months). However, platelet counts at 5 months in patients not treated with bevacizumab decreased much less than in patients who received bevacizumab. In patients not treated with bevacizumab, plasma levels of VWF:Ag increased as the number of chemotherapy cycles increased (P<0.001 at 3 months, P = 0.027 at 5 months), but there was no change in patients treated with bevacizumab (P = 0.094 at 3 months, P = 0.156 at 5 months) (Fig 1B). Plasma levels of ADAMTS13:AC were unchanged in both groups (Fig 1C). Serum AST levels increased with the number of chemotherapy cycles in patients not treated with bevacizumab (P = 0.002 at 3 months, P = 0.013 at 5 months), but were unchanged in patients treated with bevacizumab (Fig 1D). Plasma levels of T-Bil did not change significantly in both groups, but T-Bil levels increased with the number of chemotherapy cycles in patients not treated with bevacizumab, although this difference was not statistically significantly (P = 0.083 at 3 months, P = 0.181 at 5 months) (Fig 1E). A statistical analysis between patients who received or did not receive bevacizumab on each month was performed as shown in S1 Table. Plasma VWF:Ag levels at 2 to 5 months of patients without bevacizumab were significantly lower than those with bevacizumab. In addition, serum AST levels at 3 months of patients who received becacizumab were significantly lower than those who did not received bevacizumab. Consequently, 6 of 17 patients not treated with bevacizumab developed CALI, and there were no cases of CALI among patients treated with bevacizumab.

### Comparisons between patients with and without splenomegaly

We analyzed 23 patients with CRC, as shown in Table 1. The spleen size ratio ranged from 0.83 to 2.93 (median, 1.19). Splenomegaly was found in 9 patients (39.1%), who did not have any findings of liver cirrhosis by blood test and CT image. All cases of splenomegaly occurred in patients treated with oxaliplatin-based chemotherapy without bevacizumab (Patients 9–17 in Table 1). On the other hand, no patients who received an oxaliplatin-based regimen with bevacizumab (Patients 18–23) had splenomegaly; the spleen size ratio ranged from 1.00 to 1.37 (median 1.09).

**Fig 2:**
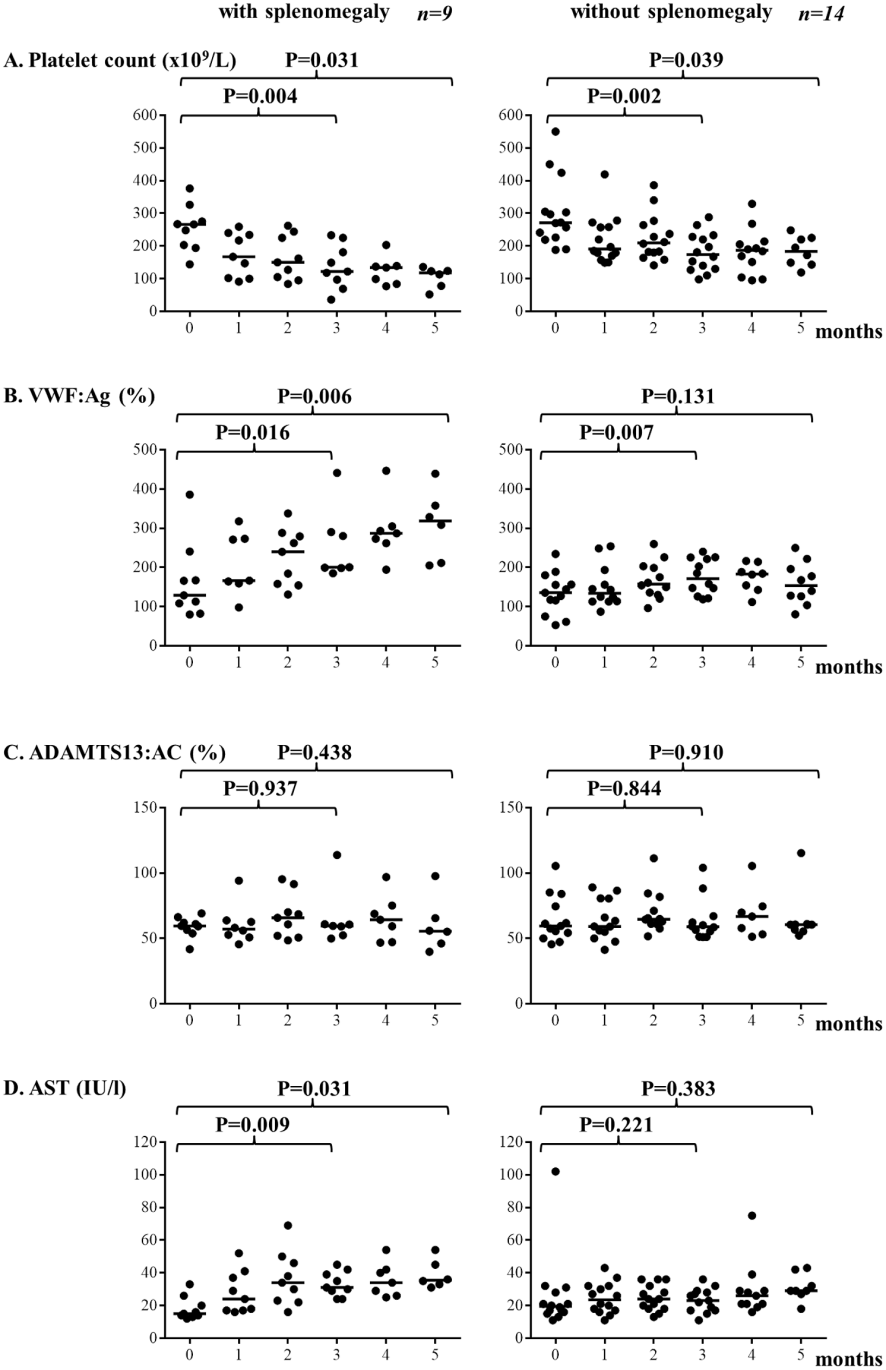
Comparison of platelet count, VWF:Ag, ADAMTS13:AC, and AST between patients with splenomegaly and those without splenomegaly. (A) Platelet counts decreased as the number of chemotherapy cycles increased in patients with and without splenomegaly. (B) Plasma levels of VWF antigen (:Ag) increased as the number of chemotherapy cycles increased in patients with splenomegaly. (C) Plasma levels of ADAMTS13:AC were unchanged in both groups. (D) Plasma levels of aspartate transaminase (AST) increased as the number of chemotherapy cycles increased in patients with splenomegaly, but not in patients without splenomegaly. VWF:Ag von Willebrand factor antigen, ADAMTS13:AC ADAMTS13 activity, AST Aspartate transaminase.

We classified the 23 patients into 2 categories: those with splenomegaly (n = 9) and without splenomegaly (n = 14). As shown in Fig 2A, platelet count decreased as the number of chemotherapy increased in patients with splenomegaly (P = 0.004 at 3 months, P = 0.031 at 5 months) and without splenomegaly (P = 0.002 at 3 months, P = 0.039 at 5 months). These findings are related to bone marrow suppression as a result of chemotherapy. However, thrombocytopenia at 5 months was more pronounced in patients with splenomegaly than in those without splenomegaly (P = 0.005, S2 Table). Plasma levels of VWF:Ag increased with the number of chemotherapy among patients with splenomegaly (P = 0.016 at 3 months, P = 0.006 at 5 months), but not among patients without splenomegaly (Fig 2B, S2 Table). As shown in Fig 2C, plasma levels of ADAMTS13:AC did not change significantly in both groups. Plasma levels of AST increased as the number of chemotherapy cycles increased in patients with splenomegaly (P = 0.009 at 3 months, P = 0.031 at 5 months), but not in patients without splenomegaly (Fig 2D). A statistical analysis between patients with and without splenomegaly on each month was performed as shown in S2 Table. Platelet counts at 2 and 5 months of patients with splenomegaly were significantly lower than those of patients without splenomegaly. Plasma VWF:Ag levels at 2, 4 and 5 months of patients with splenomegaly were significantly higher than those of patients without splenomegaly. Serum AST level at 3 months of patients with splenomegaly were significantly higher than those of patients without splenomegaly.

### VWF multimer analysis in patients who did not receive bevacizumab

#### i) Patients who developed CALI

**Fig 3:**
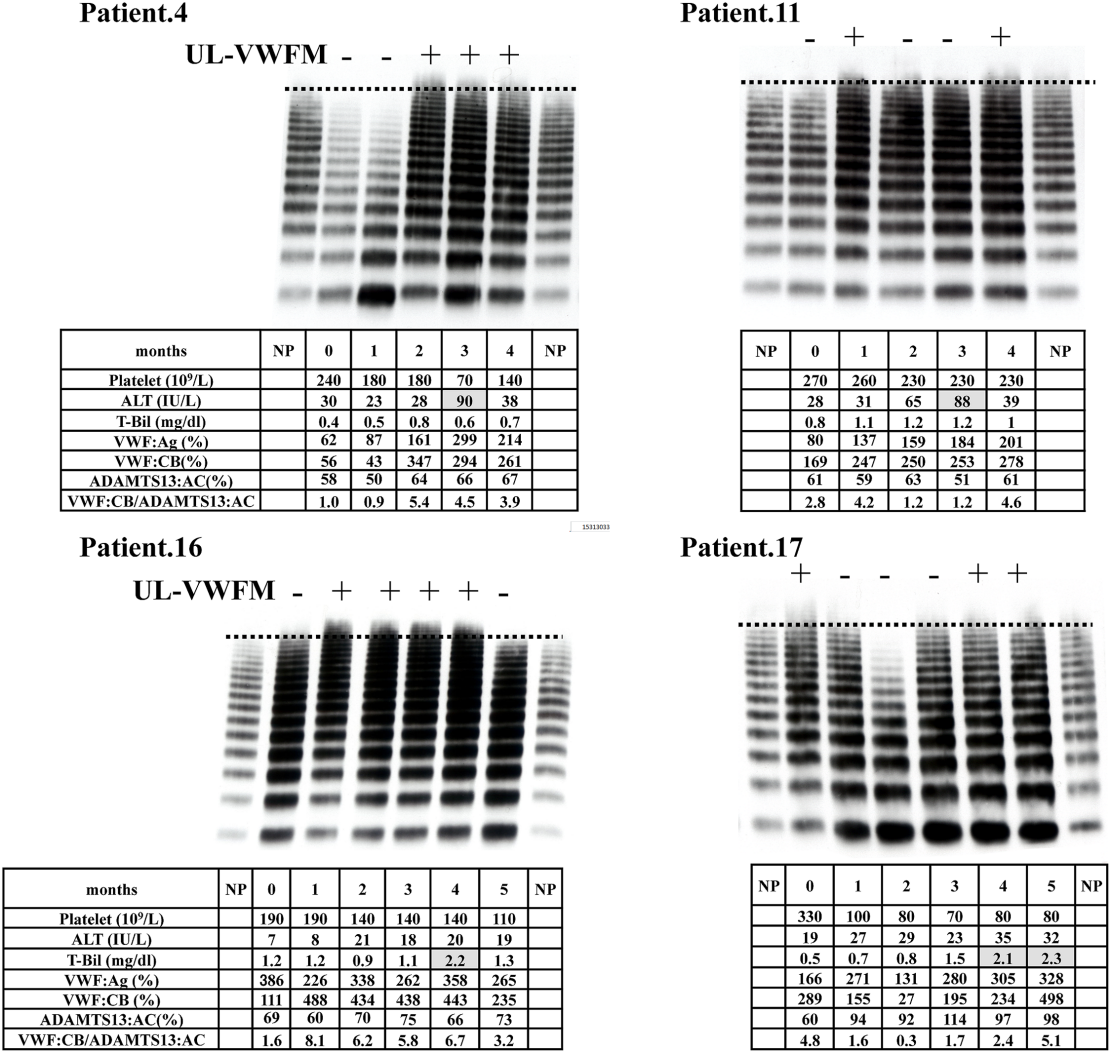
VWF multimer analysis in patients with CALI who were not treated with bevacizumab. VWF multimer analysis was performed in 4 representative patients out of 6 patients with CALI not treated with bevacizumab. These patients developed CALI during month 3 or 4. UL-VWFMs were found before and during CALI in all patients. Decreased levels of H-VWFMs were found in Patient 4 at months 0 and 1, and in Patient 17 at month 2. VWF von Willebrand factor, CALI chemotherapy-associated liver injury, UL-VWFMs unusually-large VWF multimers, H-VWFM high molecular weight VWF multimers, AST aspartate transaminase, T-Bil total bilirubin, VWF:Ag VWF antigen, VWF:CB VWF collagen binding activity.

We performed VWF multimer analysis in 4 representative patients out of 6 patients with CALI who were not treated with bevacizumab (Fig 3). Patient 4 developed CALI in 3 months after starting chemotherapy. Platelet count dropped sharply to 70×109/L in month 3, when CALI developed. H-VWFM levels were decreased in months 0 and 1. UL-VWFMs appeared in months 2, 3, and 4. Plasma VWF:Ag levels were relatively low in months 0 and 1, and increased up to 299% in month 3. Plasma levels of ADAMTS13:AC were within the normal range (>50% of normal). VWF:CB levels and the ratio of VWF:CB to ADAMTS13:AC was sharply increased after month 2 at the same time as UL-VWFM appeared. There was no instances of splenomegaly after the start of chemotherapy. Patient 11 was diagnosed with CALI in month 3. Platelet counts remained in the normal range. Plasma levels of VWF:Ag gradually increased along with the number of chemotherapy cycles. UL-VWFMs were found in months 1 and 5, when the ratios of VWF:CB to ADAMTS13:AC were clearly high values. He developed splenomegaly during chemotherapy. Patient 16 was diagnosed with CALI in month 4. Platelet count gradually decreased along with the number of chemotherapy cycles. He had extremely high VWF:Ag levels even in month 0, but VWF:CB level was normal and UL-VWFMs were not detected at this point. Both VWF:Ag and VWF:CB levels remained continuously high and UL-VWFMs were found between months 1 and 4. Splenomegaly was observed in this patient. Patient 17 developed CALI in month 4. His platelet count decreased during chemotherapy. Both VWF:Ag and VWF:CB levels were relatively high, with UL-VWFMs detected in month 0. His VWF:Ag level increased but VWF:CB decreased during month 1 without UL-VWFMs. However, in month 2 both VWF levels suddenly dropped and H-VWFMs disappeared. Subsequently, H-VWFM levels increased and UL-VWFMs were found in months 4 and 5. This patient developed splenomegaly during chemotherapy. All 4 patients who developed CALI had UL-VWFMs before and at the time of CALI diagnosis.

#### ii) Patients who did not develop CALI

**Fig 4:**
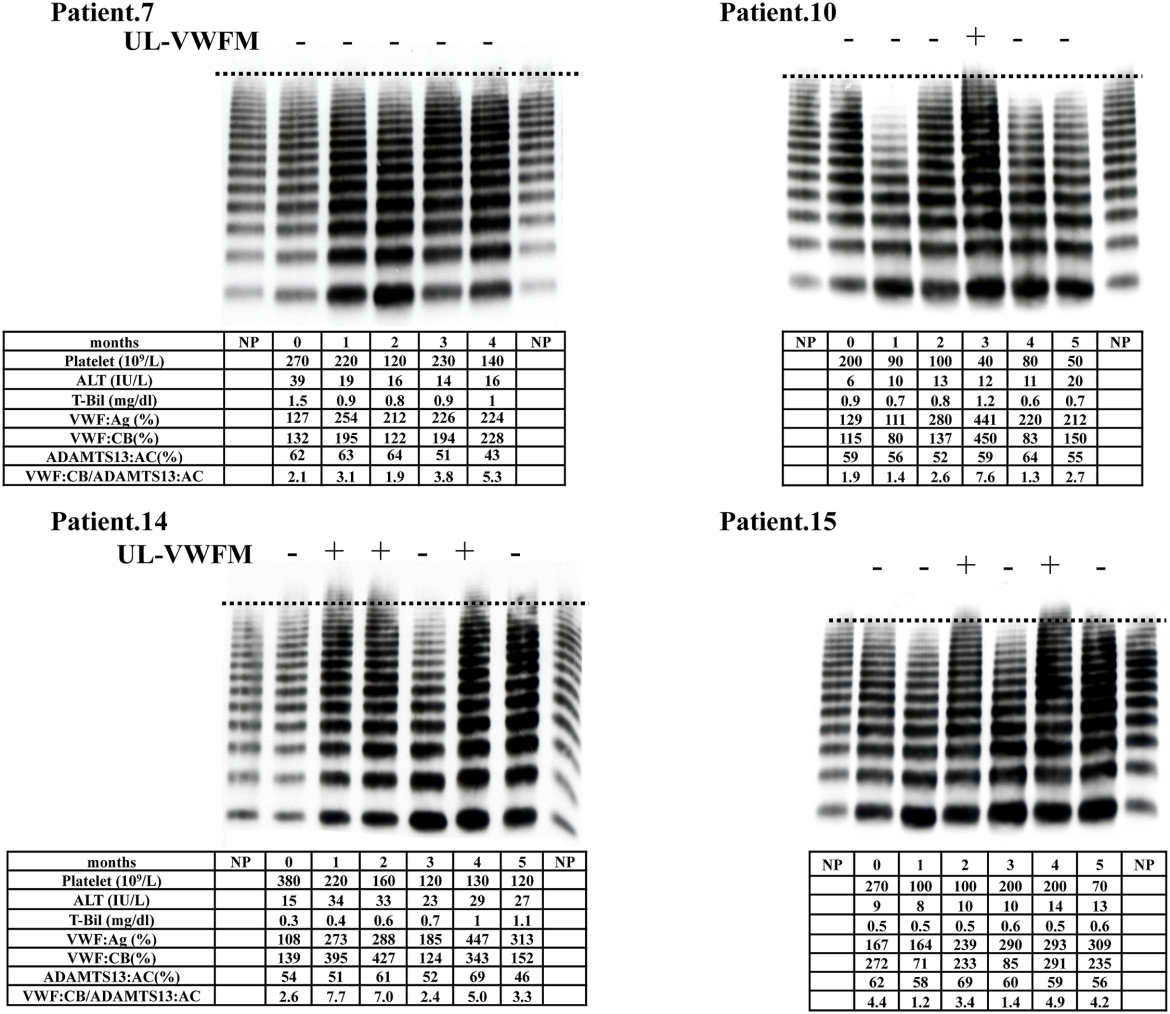
VWF multimer analysis in patients without CALI who were not treated with bevacizumab. Results of VWF multimer analysis in 4 representative patients out of 11 patients without CALI not treated with bevacizumab are shown. UL-VWFMs were found in Patients 10, 14, and 15, who did not develop CALI. In Patient 10, decreased levels of H-VWFMs were observed during months 1 and 4. VWF von Willebrand factor, CALI chemotherapy-associated liver injury, UL-VWFMs unusually-large VWF multimers, H-VWFM high molecular weight VWF multimers, AST aspartate transaminase, T-Bil total bilirubin, VWF:Ag VWF antigen, VWF:CB VWF collagen binding activity.

Results from VWF multimer analysis in 4 representative patients out of 11 patients without CALI who were not treated with bevacizumab are shown in Fig 4. In Patient 7, his platelet count dropped slightly in months 2 and 4. Plasma levels of VWF:Ag increased after the start of chemotherapy. However, UL-VWFMs were not found during chemotherapy. He did not develop splenomegaly after chemotherapy. In Patient 10, his platelet count was low during the start of chemotherapy. He developed splenomegaly after chemotherapy. VWF:Ag levels increased 2 months after starting chemotherapy. VWF multimer analysis showed lower levels of H-VWFs in month 1, which subsequently increased. UL-VWFMs were found in month 3, but they decreased again in months 4 and 5. The change of VWF:CB levels paralleled with the levels of H-VWF and UL-VWFM. He developed splenomegaly after chemotherapy. In Patient 14, platelet counts decreased gradually with the number of chemotherapy cycles. Both levels of VWF:Ag and VWF:CB were elevated after the start of chemotherapy. UL-VWFMs were found in months 1, 2, 4 and 5. In Patient 15, platelet count decreased in months 1, 2, and 5. Plasma VWF:Ag levels increased after month 2. UL-VWFMs were found in months 2 and 4 with the elevation of VWF:CB. Splenomegaly was observed in this patient. In this group, UL-VWFMs were found during chemotherapy in patients with splenomegaly, except for Patient 7.

### VWF multimer analysis in patients treated with bevacizumab

S1 Fig shows the VWF multimer analysis results in 4 representative patients out of 6 patients treated with bevacizumab. No patient who received bevacizumab developed both CALI and splenomegaly during chemotherapy. The platelet count in these patients was maintained at almost normal levels. All 4 patients had nearly normal levels of VWF:Ag. Moreover, no patients had any apparent abnormalities in VWF multimer distribution, including the lack of H-VWFMs and the appearance of UL-VWFMs. However, VWF:CB levels were relatively high compared with VWF:Ag levels.

### Histopathological evidence of sinusoidal obstruction in the liver

**Fig 5:**
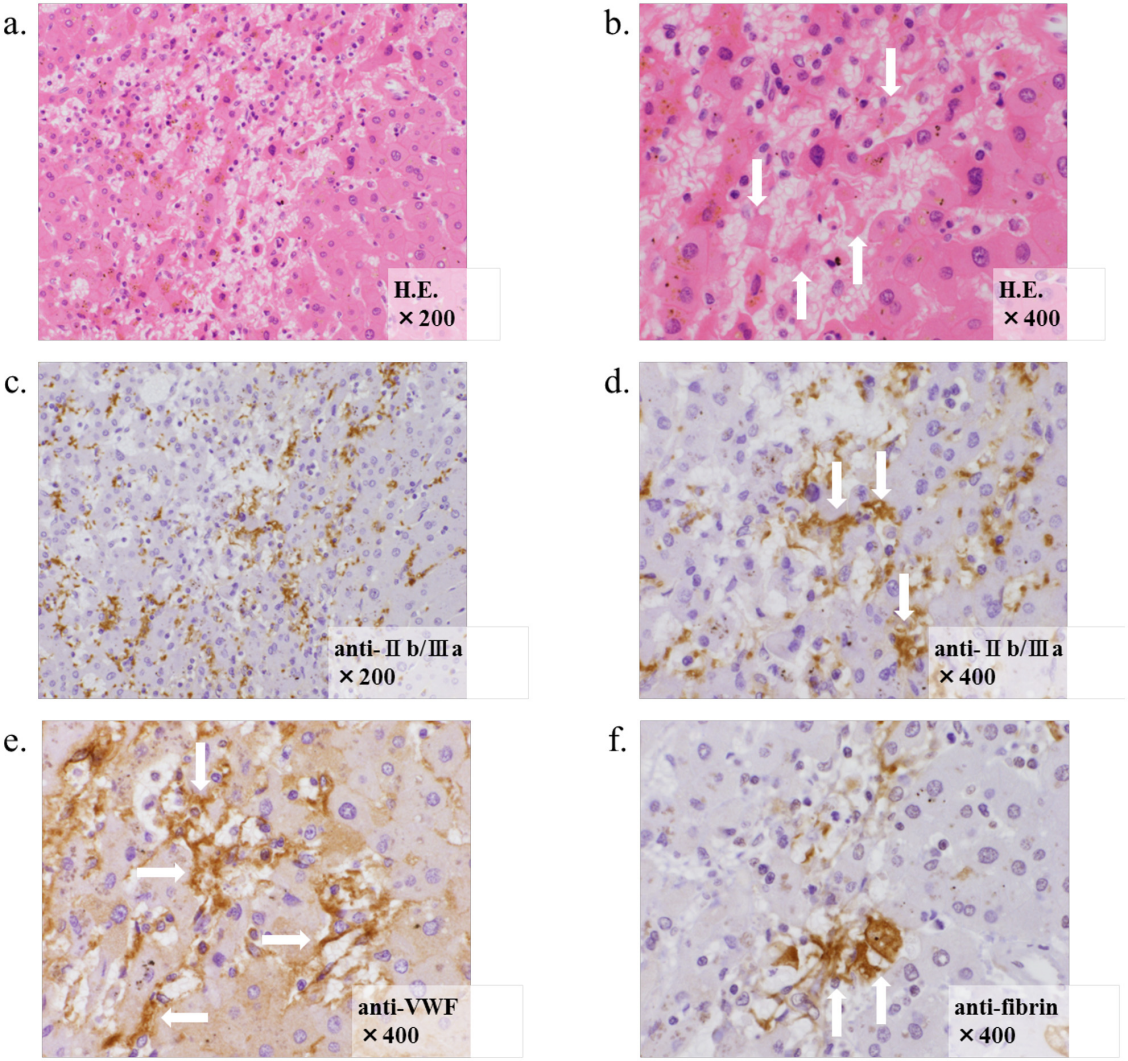
Immunohistochemical analysis of liver specimens in patients with SOS. Histological findings in the liver with hematoxylin and eosin (H. E.) staining in Patient 4 included extensive Grade 2 sinusoidal dilatation (A) and platelet thrombi in the liver sinusoids, as indicated with white arrows (B). Many of these thrombi were positive for both platelet-specific anti-IIb/IIIa (C, D) and anti-VWF (E), which showed that they are platelet thrombi, as indicated with arrows. Some thrombi were positive for fibrinogen (F), which indicated that they are fibrinogen thrombi, but there were much less frequently observed than platelet thrombi.

We performed liver sections to evaluate for metastatic liver injury during chemotherapy in 4 patients. One patient had Grade 0 sinusoidal congestion, one patient had Grade 1, and 2 patients had Grade 2. Consecutive slices in the same patient immunohistochemically stained with platelet-specific anti-IIb/IIIa, anti-VWF, anti-fibrin antibodies demonstrated Grade 1 and 2 SOS. As shown in Fig 5a and 5b, Patient 4’s liver tissue demonstrated extensive Grade 2 sinusoidal dilatation and platelet thrombi in the liver sinusoids. Many of these thrombi were positive for both IIb/IIIa and VWF (Fig 5d and 5e), indicating that they were platelet thrombi. Some thrombi were positive for fibrin (Fig 5f). These results indicated that sinusoidal congestion mainly resulted from platelet thrombi in the liver sinusoids.

## Discussion

The introduction of oxaliplatin-based chemotherapy contributed to a significant improvement in prognosis among patients with CRC. However, liver injury, including SOS, has been observed after chemotherapy containing oxaliplatin, which is called “blue liver” by hepatic surgeons [22]. The precise mechanism of liver injury due to oxaliplatin-based chemotherapy remains unclear. However, oxaliplatin is more toxic to sinusoidal endothelial cells than hepatocytes [23]. In addition, increases in spleen size and decreases in platelet count were commonly observed in patients with CRC on oxaliplatin-based chemotherapy [8].

Two hypotheses have been proposed to explain the mechanism underlying oxaliplatin-induced thrombocytopenia [7, 8]. One is bone marrow suppression by chemotherapy and the other is splenic sequestration of platelets secondary to portal hypertension. The first hypothesis is not specific for oxaliplatin and cannot explain the association among thrombocytopenia, SOS and splenomegaly. We found differences in the extent of platelet count decreases after chemotherapy between patients who received and did not receive bevacizumab (Fig 1A). Bevacizumab did not block myelosuppression associated with chemotherapy. The second mechanism could explain the relationship between splenomegaly and thrombocytopenia in addition to SOS. Based on our previous study of patients with SOS after SCT, we hypothesize that the second mechanism is related to VWF-rich platelet thrombosis in sinusoids, which induces portal hypertension and splenomegaly.

To the best of our knowledge, this is the first study analyzing the levels of VWF:Ag, activity and the appearance of UL-VWFMs in CRC patients with CALI from oxaliplatin-based chemotherapy. In this study, we chose VWF:CB as VWF activity, since this method appear to be reproducible and sensitive. Collagen binding assay is based on the physiological principle of the interaction between VWF and collagen. The adhesive activity of VWF depends the molecular size of VWF [24]. Therefore, VWF:CB usually increase with the molecular size of VWF. As shown in Figs 3 and 4, VWF:CB levels showed good correlation with the appearance of UL-VWFM. However, as shown in S1 Fig, VWF:CB levels were high even in the plasmas with the lack of UL-VWFM. These observations that high level of VWF:CB was found even in the sample with normal VWF multimers were reported in previous study [25]. This might be because the evaluation of VWF activity was difficult in one method.

Based on the results of this study, we speculate that thrombocytopenia is mainly caused by platelet consumption in platelet thrombi, and splenomegaly is caused by the occlusion of liver sinusoids.

SOS is a well-known life-threatening complication of SCT, which is clinically diagnosed by the triad of hepatomegaly, ascites, and hyperbilirubinemia [26]. It is histologically characterized by sinusoidal dilatation, congestion, and nodular regenerative hyperplasia [27]. The sinusoidal endothelial cell is also suspected to be the primary site of toxic injury from chemotherapy and/or radiation before SCT. Severe endothelial cell damage results in the release of UL-VWFMs from endothelial cells [14]. We have reported that levels of high to intermediate VWFMs were decreased during the early post-SCT phase, but UL-VWFMs appeared just before VOD onset [13]. The most important function of VWF is to act as the molecular glue for platelet adhesion and aggregation at sites of vascular injury. VWF binding to platelets through glycoprotein 1b depends on its molecular weight. Therefore, UL-VWFMs are the most active form for platelet thrombus formation. VWF is exclusively produced in endothelial cells and stored in Weibel-Palade bodies (WPBs) in endothelial cells [28]. In response to a variety of agonists such as thrombin, histamine, VEGF, serotonin, epinephrine, and vasopressin, VWF is secreted into the circulation [29]. The vasopressin analogue desmopressin is used to increase plasma VWF levels in the treatment of von Willebrand disease [30]. There are 2 mechanisms of VWF secretion from endothelial cells. One is release from severely injured endothelial cells, and the other is exocytosis of WPBs by endothelial cells stimulated by various cytokines.

In this study, plasma levels of ADAMTS13:AC were not decreased (>50% of normal) during oxaliplatin-based chemotherapy. We have reported that plasma levels of ADAMTS13:AC were decreased in patients with SCT-associated SOS [12]. The lowest values of ADAMTS13:AC were found 14 days after SCT [12, 13]. ADAMTS13 is exclusively synthesized by stellate cells in the liver [31]. Chemotherapy and/or radiation associated with SCT damages stellate cells, resulting decreased ADAMTS13:AC. However, oxaliplatin-based chemotherapy might cause much less damage to stellate cells in the liver. To study short-term changes in factors, monthly examinations might be insufficient. Therefore, we could not precisely characterize changes in plasma ADAMTS13 activity levels.

Ribero et al [11] reported that oxaliplatin plus bevacizumab was oncologically more effective than oxaliplatin alone and the incidence of hepatic injury was lower when bevacizumab was used with oxaliplatin. The effects of bevacizumab against tumors include the regulation of angiogenesis and improved delivery of chemotherapy [9]. However, it is unclear how bevacizumab protects from hepatic injury in patients treated with oxaliplatin-based chemotherapy. VEGF is reported to activate endothelial exocytosis of WPBs, which leads to the release of VWF from endothelial cell [32]. Recently, VEGF was identified as a strong promoter of endothelial cell activation accompanied by UL-VWFM release in tumor microvessels [33]. We speculated that VEGF might be one of the causative factors for CALI and SOS after oxaliplatin-based chemotherapy. In our patients, VWF levels might increase due to both endothelial cell activation by VEGF and the sinusoidal endothelial cell damage by oxaliplatin-based chemotherapy. Therefore, VWF levels together with UL-VWFM increase in blood circulation especially in sinusoids. As consequence, VWF-rich platelet thrombi was made in sinusoids and resulted in the development of CALI and SOS. Bevacizumab, an anti-VEGF monoclonal antibody, reduces plasma levels of VEGF, and thereby lowers plasma levels of VWF to some extent as shown in Fig 1 and S1 Table. In fact, one study reported that plasma VEGF levels before a conditioning regimen were significantly higher in patients with SCT-associated SOS than in patients without SOS [34].

Although this study used a novel approach to study liver injury due to oxaliplatin-based chemotherapy in patients with CRC, there are some limitations. First, the number of patients analyzed in this study was relatively small. Therefore, sufficient statistical analysis was not possible. Second, the blood samples were collected once a month and prior to the administration of chemotherapy. Since the factors analyzed in this study seemed to change over a short period of time, we might have missed the correct sequence of change in these factors. Finally, pathological examination of liver tissue to diagnose SOS was performed only in 4 patients. Of these, 2 patients had evidence of Grade 2 SOS (Patients 4 and 7). Patient 4 developed CALI and SOS confirmed by the presence of VWF-rich platelet thrombi in the sinusoids (Fig 5). This is regarded as a convincing cause of CALI. However, Patient 7 had Grade 2 SOS, but not CALI. This might have been due to the timing of blood samples or different pathophysiological mechanisms for CALI and SOS. Conversely, one patient (Patient 11) had CALI, but not SOS. This might be due to the timing of liver resection or patchy SOS findings in the part of the liver examined. It would be necessary to perform pathological examinations in all patients treated with oxaliplatin-based chemotherapy to confirm the existence of SOS.

In conclusion, we found an association between VWF and liver injury, including SOS, in CRC patients treated with oxaliplatin-based chemotherapy. VWF-rich platelet thrombi in liver sinusoids due to UL-VWFMs secreted as a result of endothelial injury might be one cause of CALI associated with oxaliplatin-based chemotherapy. Bevacizumab could protect against CALI associated with oxaliplatin-based chemotherapy through lowering plasma levels of VWF.

## Supporting Information

S1 Fig VWF multimer analysis in patients treated with bevacizumab. VWF multimer analysis was performed in 4 representative patients out of 6 patients treated with bevacizumab. None of the patients developed both CALI and splenomegaly during chemotherapy. All 4 patients had nearly normal levels of VWF:Ag. UL-VWFMs were not observed except at month 0 in Patient 22. VWF von Willebrand factor, CALI chemotherapy-associated liver injury, UL-VWFMs unusually-large VWF multimers, H-VWFM high molecular weight VWF multimers, AST aspartate transaminase, T-Bil total bilirubin, VWF:Ag VWF antigen, VWF:CB VWF collagen binding activity.
(TIF)

S1 Table Comparison between patients who received or did not receive bevacizumab. (DOCX)

S2 Table Comparisons between patients with and without splenomegaly. (DOCX)
