# Supplementary figures and images for: von Willebrand Factor-Rich Platelet Thrombi in the Liver Cause Sinusoidal Obstruction Syndrome following Oxaliplatin-Based Chemotherapy
Source: PLoS One. 2015 Nov 18;10(11):e0143136. doi: 10.1371/journal.pone.0143136 (PMC4651512; doi:10.1371/journal.pone.0143136)

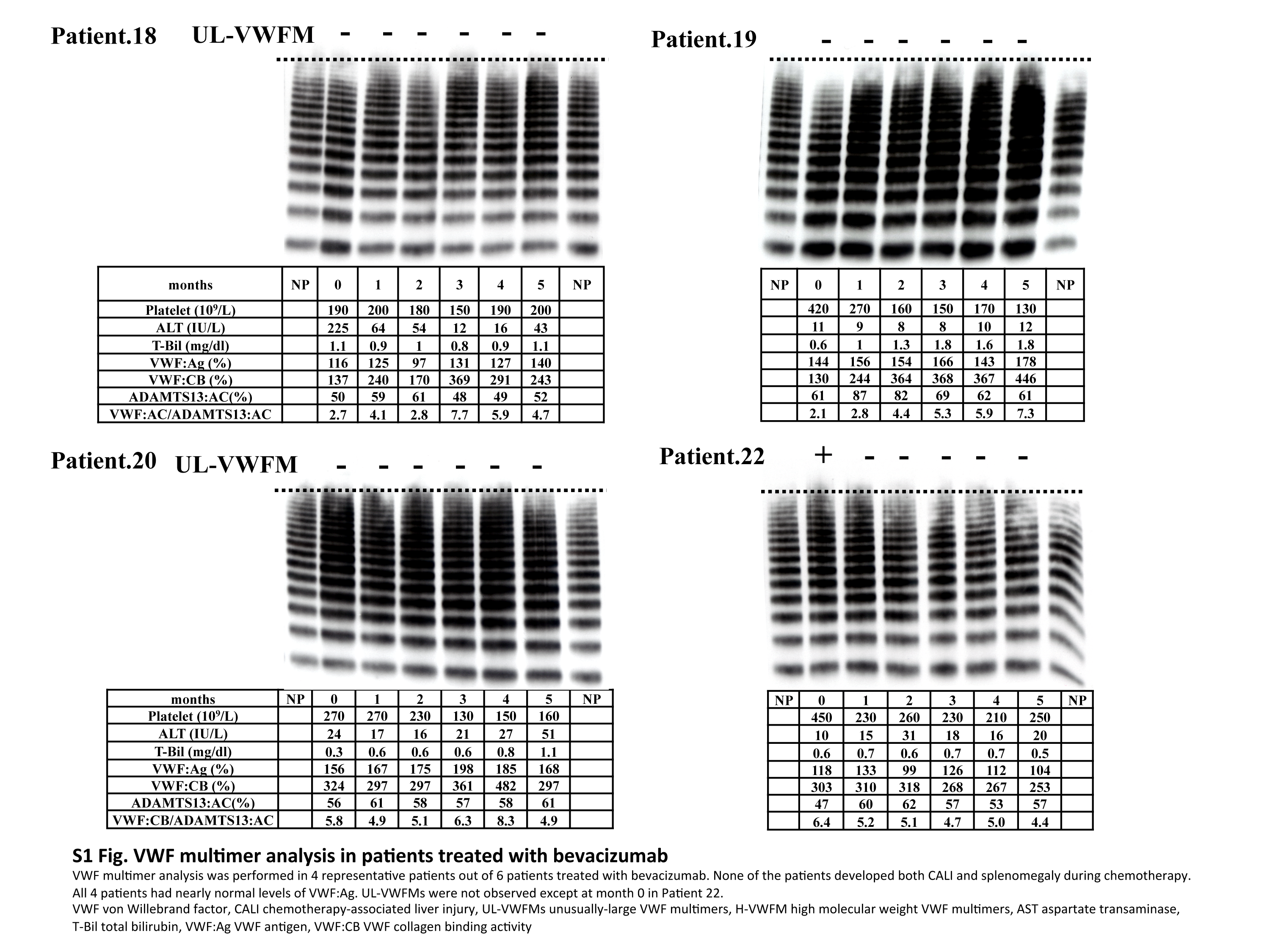

Supplement: S1 Fig — VWF multimer analysis was performed in 4 representative patients out of 6 patients treated with bevacizumab. None of the patients developed both CALI and splenomegaly during chemotherapy. All 4 patients had nearly normal levels of VWF:Ag. UL-VWFMs were not observed except at month 0 in Patient 22. VWF von Willebrand factor, CALI chemotherapy-associated liver injury, UL-VWFMs unusually-large VWF multimers, H-VWFM high molecular weight VWF multimers, AST aspartate transaminase, T-Bil total bilirubin, VWF:Ag VWF antigen, VWF:CB VWF collagen binding activity. (TIF) [file pone.0143136.s001.tif]
