# Supplementary material for: von Willebrand Factor-Rich Platelet Thrombi in the Liver Cause Sinusoidal Obstruction Syndrome following Oxaliplatin-Based Chemotherapy
Source: PLoS One. 2015 Nov 18;10(11):e0143136. doi: 10.1371/journal.pone.0143136 (PMC4651512; doi:10.1371/journal.pone.0143136)
Supplement: S1 Table — (DOCX) [file pone.0143136.s002.docx]

S1 Table 1. Comparison between patients who received or did not receive bevacizumab.

|  | month | with bevacizumab | without bevacizumab | P |
| --- | --- | --- | --- | --- |
| **Platelet count (x10^9^/L)** | 0 | 287 (188-450) | 262 (144-376) | 0.708 |
|  | 1 | 188 (157-172) | 182 (91-419) | 0.674 |
|  | 2 | 196 (141-277) | 183 (84-386) | 0.865 |
|  | 3 | 160 (110-233) | 140 (36-288) | 0.861 |
|  | 4 | 183 (95-214) | 134 (77-329) | 0.500 |
|  | 5 | 172 (119-248) | 130 (52-225) | 0.304 |
| **VWF:Ag (%)** | 0 | 131 (75-189) | 136 (53-386) | 0.865 |
|  | 1 | 125 (113-194) | 162 (87-318) | 0.207 |
|  | 2 | 142 (97-199) | 194 (131-338) | 0.027 |
|  | 3 | 153 (119-203) | 222 (122-441) | 0.017 |
|  | 4 | 143 (112-185) | 239 (155-447) | 0.018 |
|  | 5 | 154 (81-250) | 217 (127-439) | 0.045 |
| **VWF:CB (%)** | 0 | 185 (115-354) | 271 (56-369) | 0.914 |
|  | 1 | 302 (80-310) | 247 (43-943) | 0.915 |
|  | 2 | 308 (137-369) | 336 (92-768) | 0.685 |
|  | 3 | 348 (268-450) | 315 (85-663) | 0.825 |
|  | 4 | 279 (83-482) | 318 (83-803) | 0.689 |
|  | 5 | 197 (150-243) | 235 (98-676) | 0.474 |
| **ADAMTS13:AC (%)** | 0 | 58 (47-75) | 61 (42-85) | 0.473 |
|  | 1 | 59 (48-87) | 57 (38-94) | 0.555 |
|  | 2 | 62 (58-82) | 65 (36-95) | 0.971 |
|  | 3 | 58 (51-88) | 60 (50-67) | 0.701 |
|  | 4 | 58 (53-75) | 67 (47-97) | 0.769 |
|  | 5 | 57 (52-61) | 61 (40-98) | 0.500 |
| **AST (IU/l)** | 0 | 19 (15-21) | 17 (11-33) | 0.483 |
|  | 1 | 19 (14-43) | 26 (11-52) | 0.362 |
|  | 2 | 24 (15-36) | 28 (13-69) | 0.441 |
|  | 3 | 20 (15-27) | 30 (11-45) | 0.009 |
|  | 4 | 21 (16-29) | 29 (19-75) | 0.054 |
|  | 5 | 29 (18-43) | 33 (29-54) | 0.349 |
| **T-Bil (mg/dl)** | 0 | 0.7 (0.3-1.1) | 0.5 (0.2-1.5) | 0.916 |
|  | 1 | 0.8 (0.4-1.1) | 0.7 (0.2-2.0) | 0.752 |
|  | 2 | 0.8 (0.4-1.8) | 0.8 (0.2-1.4) | 0.698 |
|  | 3 | 0.8 (0.5-1.8) | 0.9 (0.3-1.5) | 0.911 |
|  | 4 | 0.8 (0.5-1.0) | 0.9 (0.5-1.1) | 0.456 |
|  | 5 | 0.7 (0.5-1.1) | 0.9 (0.5-1.6) | 0.286 |

median (minimum-maximum)

VWF:Ag VWF antigen, VWF:CB VWF collagen binding activity, ADAMTS13:AC ADAMTS13 activity

AST aspartate transaminase, T-Bil total bilirubin
