# Supplementary material for: von Willebrand Factor-Rich Platelet Thrombi in the Liver Cause Sinusoidal Obstruction Syndrome following Oxaliplatin-Based Chemotherapy
Source: PLoS One. 2015 Nov 18;10(11):e0143136. doi: 10.1371/journal.pone.0143136 (PMC4651512; doi:10.1371/journal.pone.0143136)
Supplement: S2 Table — (DOCX) [file pone.0143136.s003.docx]

S2 Table. Comparisons between patinets with and without splenomegaly.

|  | month | with splenomegaly | without splenomegaly | P |
| --- | --- | --- | --- | --- |
| **Platelet count (x10^9^/L)** | 0 | 266 (144-376) | 271 (188-450) | 0.477 |
|  | 1 | 167 (91-259) | 191 (149-419) | 0.156 |
|  | 2 | 150 (84-262) | 210 (141-386) | 0.046 |
|  | 3 | 122 (36-233) | 174 (98-288) | 0.123 |
|  | 4 | 134 (77-203) | 187 (95-329) | 0.056 |
|  | 5 | 118 (52-136) | 184 (119-248) | 0.005 |
| **VWF:Ag (%)** | 0 | 129 (80-386) | 136 (53-234) | 0.781 |
|  | 1 | 167 (98-318) | 134 (87-254) | 0.056 |
|  | 2 | 240 (131-338) | 158 (97-260) | 0.043 |
|  | 3 | 201 (185-290) | 172 (119-240) | 0.068 |
|  | 4 | 287 (194-305) | 183 (112-217) | 0.001 |
|  | 5 | 318 (205-358) | 154 (81-250) | 0.003 |
| **VWF:CB (%)** | 0 | 169 (60-369) | 269 (56-354) | 0.744 |
|  | 1 | 247 (71-943) | 302 (43-858) | 0.980 |
|  | 2 | 250 (92-434) | 347 (122-768) | 0.285 |
|  | 3 | 314 (85-663) | 334 (194-586) | 0.841 |
|  | 4 | 278 (83-680) | 318 (83-803) | 0.457 |
|  | 5 | 194 (98-676) | 330 (150-589) | 0.082 |
| **ADAMTS13:AC (%)** | 0 | 60 (42-69) | 60 (46-85) | 0.781 |
|  | 1 | 57 (46-94) | 59 (41-89) | 0.838 |
|  | 2 | 66 (49-95) | 65 (52-84) | 1.000 |
|  | 3 | 60 (50-61) | 59 (51-88) | 0.902 |
|  | 4 | 64 (47-97) | 67 (51-75) | 0.805 |
|  | 5 | 56 (40-98) | 61 (52-61) | 0.376 |
| **AST (IU/l)** | 0 | 15 (12-33) | 21 (11-32) | 0.219 |
|  | 1 | 24 (16-52) | 24 (11-43) | 0.705 |
|  | 2 | 34 (16-69) | 24 (13-36) | 0.083 |
|  | 3 | 31 (24-45) | 23 (11-36) | 0.010 |
|  | 4 | 34 (25-54) | 26 (16-75) | 0.092 |
|  | 5 | 36 (31-54) | 29 (18-43) | 0.060 |

median (minimum-maximum)

VWF:Ag VWF antigen, VWF:CB VWF collagen binding activity, ADAMTS13:AC ADAMTS13 activity

AST aspartate transaminase
